# A Detailed Questionnaire for the Evaluation of Health Management in Dairy Sheep and Goats

**DOI:** 10.3390/ani10091489

**Published:** 2020-08-24

**Authors:** Daphne T. Lianou, Ioanna P. Chatziprodromidou, Natalia G. C. Vasileiou, Charalambia K. Michael, Vasia S. Mavrogianni, Antonis P. Politis, Nikos G. Kordalis, Charalambos Billinis, Alexios Giannakopoulos, Elias Papadopoulos, Ilias Giannenas, Katerina S. Ioannidi, Angeliki I. Katsafadou, Dimitris A. Gougoulis, Delia Lacasta, Mariangela Caroprese, George C. Fthenakis

**Affiliations:** 1 Veterinary Faculty, University of Thessaly, 43100 Karditsa, Greece; dlianou@vet.uth.gr (D.T.L.); cmichail@uth.gr (C.K.M.); vmavrog@vet.uth.gr (V.S.M.); apolitis.vet@gmail.com (A.P.P.); nikolaoskordalis@gmail.com (N.G.K.); billinis@vet.uth.gr (C.B.); alexiosg@yahoo.gr (A.G.); kate_ioan@windowslive.com (K.S.I.); agkatsaf@vet.uth.gr (A.I.K.); dgoug@vet.uth.gr (D.A.G.); 2 Medical School, University of Patras, 26504 Patras, Greece; ioannachatzi@med.upatras.gr; 3 Faculty of Animal Science, University of Thessaly, 41500 Larissa, Greece; vasileiounat@gmail.com; 4 Faculty of Veterinary Medicine, Aristotle University of Thessaloniki, 54124 Thessaloniki, Greece; eliaspap@vet.auth.gr (E.P.); igiannenas@vet.auth.gr (I.G.); 5 Animal Pathology Department, Instituto Agroalimentario de Aragón-IA2 Universidad de Zaragoza-CITA, Veterinary Faculty of Zaragoza, 50013 Zaragoza, Spain; dlacasta@unizar.es; 6 Department of Sciences of Agriculture, Food and Environment (SAFE), University of Foggia, 71122 Foggia, Italy; mariangela.caroprese@unifg.it

**Keywords:** epidemiology, field study, health management, infrastructure, nutrition, production characteristics, questionnaire, survey

## Abstract

**Simple Summary:**

The objective of this work was to develop, use and present a detailed questionnaire for the evaluation of health management in dairy small ruminants. The questionnaire includes 442 questions, which cover seven sections (general, infrastructure, animals, production characteristics, health management, nutrition, human resources). The average duration of the interview for completion of the questionnaire was 64 min. The questionnaire can be used for research work in the field, to record details in the farms into the study. In accord with the needs of a particular study, the questionnaire can be extended, by adding therein more specific questions or omitting some deemed to be less important. Moreover, the questionnaire can also be used for routine monitoring purposes, as a useful means to record and maintain details of farms during clinical work.

**Abstract:**

The objective of this work was to develop, use and present a detailed questionnaire for the evaluation of health management in dairy small ruminants; it includes 442 questions organised in seven sections: general, infrastructure, animals, production characteristics, health management, nutrition, human resources. Consistency of replies was evaluated in 27 farmers, interviewed twice. Inconsistent replies were given by all farmers to 30 different questions (Cronbach’s coefficient alpha: 0.987). Then, interviews were performed in 444 farms around Greece. Mean duration of an interview was 63.6 min. Clarifications were requested by 273 farmers to 22 different questions (maximum per farmer: 8). The experience of the investigator, the primary language of farmers and asking clarifications by the farmers affected the duration of the interview. The questionnaire can be used for research work in the field, to record details in the farms under study. In accord with the needs of a particular study, it can be modified, by adding more specific questions or omitting others deemed of less importance. Moreover, it can also be used for routine monitoring purposes, as a useful means to record and maintain details of farms during clinical work. To the best of our knowledge, the questionnaire is the most extensive and detailed one available internationally for dairy small ruminants.

## 1. Introduction

Research work can be greatly facilitated by using standardised questionnaires to gather information. These can be applied in varying situations (e.g., farms in survey studies), contributing to producing comparable results, which would further allow evaluation of findings between studies. Moreover, in animal population medicine, questionnaires are important for the detailed assessment of farms (e.g., management system, infrastructure, feeding regime, production outcomes, animal populations etc.), the outcome of which can guide the subsequent efficient usage of resources (e.g., ancillary tests to be performed). To the best of our knowledge, no detailed questionnaire is available in the international literature for use in small ruminants. The objective of this work was to develop, use and present a detailed questionnaire for the evaluation of health management in dairy sheep flocks/goat herds.

## 2. Materials and Methods

### 2.1. The Questionnaire

The questionnaire includes 442 questions organised in seven sections. The full questionnaire is presented in Table S1.

### 2.2. Development

The questionnaire was developed at the Department of Obstetrics and Reproduction of the Veterinary Faculty of the University of Thessaly; the Department is also a training centre of the European College of Small Ruminant Health Management and, in past years, has performed many field projects around Greece, from which experience was drawn to set up and develop a detailed questionnaire. Members of the academic staff of the Department and field veterinarians have contributed in the development of the questionnaire. The protocols of the study were approved by the academic board of the Faculty, meeting 34/03.04.19. Further contributions were made by the academic staff of the Department of Microbiology and Parasitology of the Faculty in relation to issues of biosecurity and wildlife, as well as by academic staff of the Laboratory of Parasitology and the Laboratory of Nutrition of the Faculty of Veterinary Medicine of the Aristotle University of Thessaloniki in relation to issues of antiparasitic drugs and nutritional management, respectively. The questionnaire was originally prepared in the Greek language.

After completion of the final draft of the questionnaire, a pilot test was conducted; seven known farmers (i.e., with whom there had been a previous professional collaboration) and four unknown farmers (i.e., ones whom the authors had never met before) were interviewed, in order to better understand whether they would comprehend the questions and to evaluate the flow of the interview. After taking into account the responses and the reactions of these 11 farmers, appropriate modifications were made and the questionnaire was finalised. There were seven sections in the questionnaire, which included open, multiple-choice, dichotomous and scaling questions (Table 1).

After finalising the questionnaire, 27 farmers (specifically, the persons within the owning families of each farm, who managed and followed up the business activities and operations in the respective sheep flocks/goat herds) were interviewed, with the objective to test consistency of the replies of the farmers. These farmers were visited and interviewed twice, with an interval of two to three months in-between, in both occasions by the same investigator (DTL). Then, the answers received in the two interviews were compared. Five of the questions (no. 007: Geographical coordinates; 042, 053, 067: Dimensions of structures; 046: Orientation of the building) were not asked, because relevant data were to be collected using hand-held global positioning system Garmin units or a laser measuring tool as appropriate. Another four of the questions (001: Farm reference; 005: Date of the visit; 006: Veterinarian(s); 033: Availability of access road) also were not asked, because the answers were known before the start of the interview. Additionally to the interview, as part of a thorough evaluation of the site, another investigator (CKM), also present during each visit, recorded details regarding as many as possible of 48 questions (Appendix A), whilst the breeds of the sheep/goats in the farm (questions: 137–140) were confirmed by a European Veterinary Specialist in Small Ruminant Health Management (GCF).

### 2.3. Application

**Figure 1:**
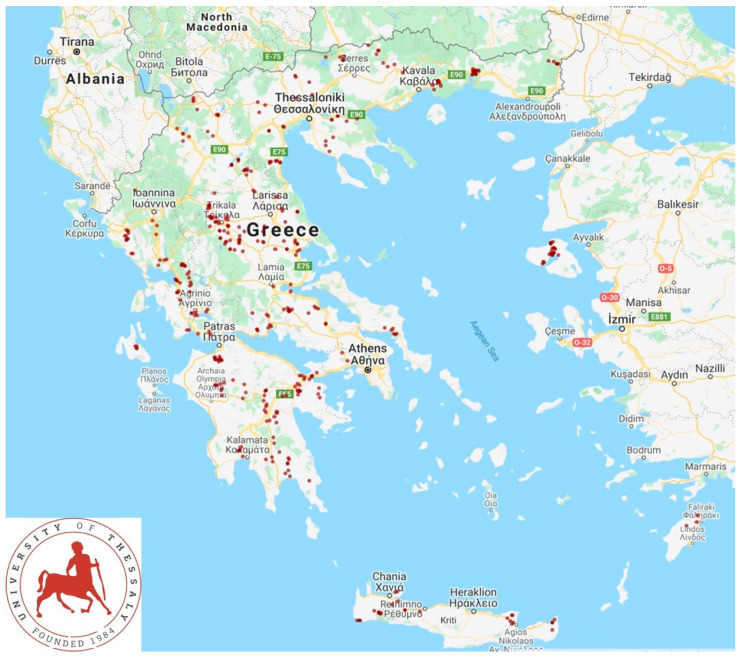
Location of 444 sheep flocks/goat herds around Greece, in which interviews were performed.

In the final stage, 444 farmers (specifically, the persons within the owning families of each farm, who managed and followed up the business activities and operations in the respective sheep flocks/goat herds) were interviewed: 325 sheep flocks and 119 goat herds in all the 13 administrative regions of Greece (Appendix B) were visited, in order to interview the respective farmers. Veterinarians active in small ruminant health management around Greece, were contacted by telephone and asked if they wished to collaborate in the investigation. In total, 48 veterinarians were contacted; of these, 47 (97.9%) agreed to collaborate. Farms were selected by the collaborating veterinarians on convenience basis (willingness of farmers to accept a visit by University personnel for an interview). Each of these veterinarians had a stable, although not contractual, association with the respective farm, among those selected for visit, and were responsible for their decisions and actions in relation to the health and welfare of the animals therein, in full accord with the relevant veterinary conduct codes [1,2]. The investigators visited all farms for the interviews. Of the 47 veterinarians, 45 accompanied the investigators during the interviews. Visits were scheduled to 446 farms, but on two occasions (0.4%), whilst the investigators had already arrived at these farms, the respective farmers mentioned that they did not want to take the interview and refused to collaborate. Details of the farms are in Table 2 and their locations round the country are shown in Figure 1.

All the interviews were performed by the same investigator (DTL). The duration of the interview was recorded. Any questions for which clarifications were requested by the respondents, were recorded. The spontaneous comments of the farmers during the interview were also recorded by the interviewing investigator (DTL), whilst the spontaneous comments of the veterinarians were recorded by another investigator (GCF).

Details regarding as many as possible of 48 questions (Appendix A) were also recorded during the on-site visit by another investigator (CKM), who was present in the farms. The breeds of the sheep/goats in the farm (questions: 137–140) were confirmed by a European Veterinary Specialist in Small Ruminant Health Management (GCF). After conclusion of the interview, details regarding as many as possible of 56 questions (Appendix C) were also collected by an investigator (DTL) from the veterinarian, who had arranged the visit.

### 2.4. Data Management and Analysis

During the development of the questionnaire, an inconsistent reply in the interviews by the two investigators was considered when the answers of a farmer to the same question differed between the two interviews; for open questions that required a quantitative answer (e.g., annual milk production in the farm, average weight of lambs at slaughter, total amount of concentrates purchased) only differences of over 5% between the two replies were taken into account. During the application of the questionnaire, the duration of the interview was calculated from the time the first question was asked to the time the final question was answered; durations were rounded to the nearest minute.

The data were entered into Microsoft Excel. Basic descriptive analysis was initially performed.

Analysis of variance or analysis of correlation, as appropriate, was performed between the number of inconsistent replies by a farmer and the animal species farmed, the management system practiced, their sex, age, farming experience, education and whether they were in full-time farming. Then, the frequency of inconsistent replies among the various types of questions (open, multiple choice, dichotomous, scaling) was evaluated in a table of cross-categorised frequency data by use of the Pearson chi-square test. The internal consistency of the answers in the repeated interviews of the 27 farmers was evaluated by calculating Cronbach’s coefficient alpha [4] in a statistical software package [5].

Differences in the duration of the interview and in the number of clarifications asked between farmers according to animal species farmed, management system practiced, sex, age, farming experience, education and whether they were full-time farmers or not, as well as the administrative region of the country, were evaluated by means of analysis of variance or analysis of correlation, as appropriate.

Linear regression of the duration of the interviews and the number of clarifications throughout the study was assessed. Also, the duration of the interview and the clarifications asked by farmers were calculated separately for the initial 222 interviews and for the last 222 interviews; then, comparisons were made by analysis of variance between the two values for each of these two measures.

In all cases, level of significance was set at *p* ≤ 0.05.

## 3. Results

### 3.1. Confirmation of the Questionnaire

Inconsistencies were found in the replies of all 27 (100%) farmers in the two interviews. Among all farmers, the median value of inconsistent replies was 6 (1.4%) per farmer (min–max = 3–10; 0.7%–2.3%). There was no association between the number of inconsistent replies by a farmer and the animal species farmed, the management system practiced, their sex, age, farming experience or education, as well as whether they were in full-time farming or not (*p* > 0.11 for all comparisons).

Inconsistencies were found in the replies to 30 different questions in the questionnaire (6.9%). The median value of inconsistent replies was 5 (1.2%) (min–max 1–14; 0.2%–3.2%) when only questions with inconsistent replies (*n* = 30) were taken into account (Appendix D). Most of these questions (76.7%) were open type questions; the proportion of inconsistent replies among open questions (12.7%) was significantly higher than that among all other types of questions (2.8%) (*p* < 0.001). 

Cronbach’s coefficient alpha was found to be 0.987.

### 3.2. Application of the Questionnaire

#### 3.2.1. Duration of the Interview

The mean value (± standard error of the mean) of the duration of the interview was 63.6 ± 0.3 min. There was evidence for a significant difference in the duration of the interview between farmers who spoke (*n* = 418) or did not speak (*n* = 26) Greek as their primary language: 63.6 ± 0.3 versus 66.0 ± 0.2 min (*p =* 0.035). There were no differences in the duration of the interview between (a) sheep or goat farmers (63.6 ± 0.3 or 64.0 ± 0.5 min, respectively; *p =* 0.50), (b) farmers practicing intensive, semi-intensive, semi-extensive or extensive management system (62.7 ± 0.8, 63.7 ± 0.4, 64.2 ± 0.5 or 63.6 ± 0.9 min, respectively; *p =* 0.467), (c) male (*n* = 414) or female (*n* = 30) farmers (63.7 ± 0.3 or 64.6 ± 0.3 min, respectively; *p =* 0.38), (d) farmers with primary (*n* = 144), secondary (*n* = 171), vocational (*n* = 69), tertiary technological (*n* = 18) or tertiary university (*n* = 42) education (63.8 ± 0.3, 64.1 ± 0.3, 64.0 ± 0.3, 61.8 ± 0.3 or 62.6 ± 0.3 min, respectively; *p =* 0.32), (e) full-time (*n* = 397) or part-time (*n* = 47) farmers (63.7 ± 0.3 or 63.5 ± 0.3 min, respectively; *p =* 0.82) and (f) farmers who employed (*n* = 157) or did not employ (*n* = 287) staff (63.3 ± 0.3 or 64.0 ± 0.3 min, respectively; *p =* 0.19). Moreover, there was no association between duration of the interview and age (*r* = −0.014) or experience (*r* = −0.034) of farmers (*p =* 0.38 or 0.23, respectively), nor between duration of the interview and the time spent by the farmer working at the farm (r = 0.013; *p =* 0.40). There was also no significant difference in the duration of the interview between farmers in the 13 administrative regions of the country (*p =* 0.28).

**Figure 2:**
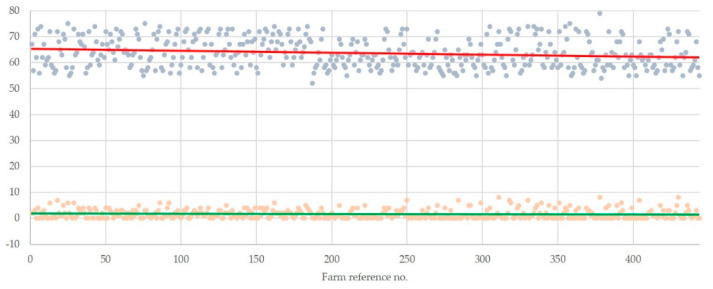
Plot diagram of the progressive duration of the interviews (fade blue spots) and clarifications asked by the farmers (fade orange spots), with slope lines: solid red line (slope: −0.0075) and solid green line (slope: −0.0006), respectively (*n* = 444) (the vertical axis shows durations of interviews (min) and no. of clarifications asked by the farmers).

The duration of the interviews decreased progressively, as the investigation advanced: slope of the duration throughout the study was −0.0075 (standard error: 5.5877) (*p =* 0.0002) (Figure 2). The mean duration of the interview was longer during the initial 222 than during the last 222 interviews: 64.8 ± 0.3 versus 62.7 ± 0.3 min (*p =* 0.0001). This was evident among farmers who spoke Greek as their primary language: 64.6 ± 0.3 versus 62.7 ± 0.3 min (*p =* 0.0007), but not among ones who did not: 66.5 ± 0.2 versus 63.0 ± 0.1 min (*p =* 0.066).

#### 3.2.2. Clarifications Asked by the Farmers

Clarifications were asked by 273 (61.5%) farmers in total (median value: one question per farmer; 0.2%) (Appendix E). There was no association between the number of clarifications asked and (a) sheep or goat farmers (1.5 ± 0.1 or 1.8 ± 0.2 questions, respectively; *p =* 0.14), (b) farmers practicing intensive, semi-intensive, semi-extensive or extensive management system (1.3 ± 0.2, 1.6 ± 0.1, 1.7 ± 0.1 or 1.6 ± 0.3 questions, respectively; *p =* 0.74), (c) male or female farmers (1.6 ± 0.1 or 2.0 ± 0.1 questions, respectively; *p =* 0.27), (d) farmers with primary, secondary, vocational, tertiary technological or tertiary university education (1.5 ± 0.1, 1.7 ± 0.1, 1.5 ± 0.1, 1.6 ± 0.1 or 1.6 ± 0.1 questions, respectively; *p =* 0.94), (e) full-time or part-time farmers (1.6 ± 0.1 or 1.8 ± 0.1 questions, respectively; *p =* 0.54) and (f) primary language of the farmers (1.6 ± 0.1 or 1.7 ± 0.1 questions, respectively; *p =* 0.90). Moreover, there was no association between the number of clarifications asked and age (*r* = −0.001) or experience (*r* = −0.036) of the farmers (*p =* 0.49 or 0.22, respectively), nor between the number of clarifications asked and the time spent by the farmer working at the farm (*r* = 0.019; *p =* 0.35). Finally, there was also no significant difference in the number of clarifications asked between farmers in the 13 administrative regions of the country (*p =* 0.85).

The number of clarifications asked by the farmers progressively did not change: slope of the clarifications asked throughout the study was −0.0006 (standard error: 1.8402) (*p =* 0.18) (Figure 2). The mean number of clarifications asked by the farmers did not differ significantly between the initial and the last 222 interviews: 1.7 ± 0.1 versus 1.5 ± 0.1 (*p =* 0.34).

Clarifications were asked in 22 different questions (5.0%) (Appendix E). Most of these questions (81.8%) were dichotomous questions; the proportion of questions for which clarifications were requested among dichotomous questions (9.3%) was significantly higher than that among all other types of questions (1.7%) (*p* < 0.001).

**Figure 3:**
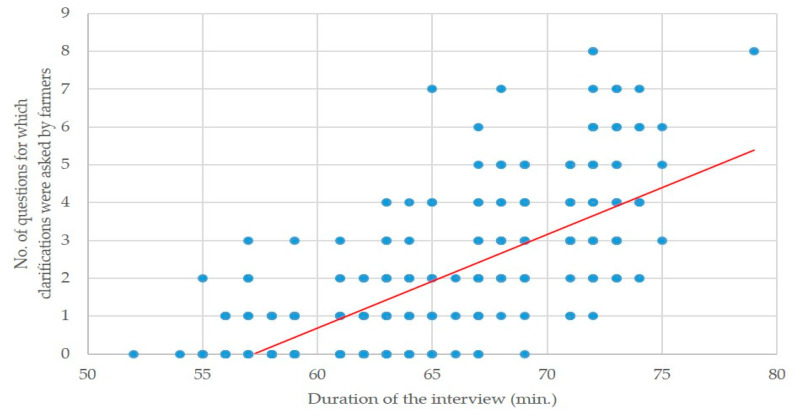
Plot diagram of the duration of the interview versus the number of clarifications asked by the farmers (solid red line indicates tendency line).

There was a clear correlation between the number of questions for which clarifications were asked by a farmer, and the respective duration of the interview (r = 0.76; *p* < 0.0001) (Figure 3).

#### 3.2.3. Qualitative Assessment by Veterinarians and Farmers

Of the 39 veterinarians from whom an opinion was acceptable (of the 47, two did not attend the interviews and six had been involved in the development of the questionnaire), 24 (61.5%) spontaneously commented about the questionnaire and all (100%) expressed a positive opinion (Table 3); of them, six (25.0%) also requested a copy of the questionnaire to use in their practices. Among the 72 (16.4% of all) farmers who spontaneously expressed an opinion, most (*n* = 51, 70.8%) indicated that the questionnaire was lengthy and time-consuming; fewer farmers expressed a positive opinion (*n* = 19, 26.4%; among them, three requested a copy of the questionnaire) or seemed annoyed with the many details asked (*n* = 2, 2.8%); in those cases, farmers were reassured by the accompanying veterinarian that the interview was performed for research purposes only and not as an official evaluation of the farm.

#### 3.2.4. Verification of Replies

The replies of the farmers were verified against the details collected by the investigators during the site visit and the details obtained from the veterinarians, who had arranged the visits. In no case were discrepancies found between the replies of the farmers and the on-site observations of the investigators or the details provided by the veterinarians.

## 4. Discussion

The study has provided information regarding a detailed questionnaire to assess the health management in sheep flocks/goat herds in farms during a countrywide investigation in Greece. Questionnaires can provide valuable information for research and clinical purposes. To the best of our knowledge, the presented questionnaire is the most extensive and detailed one available for small ruminant farms in the international literature. The questionnaire is directed mainly for dairy farms, but, nevertheless, many of its questions can be easily applied during investigations in other production systems.

In previous reports, fewer questions were included in the relevant questionnaires, which were designed according to the specific needs of the research study performed. For example, in relation to work in sheep or goats, Lafi et al. [6] have used a 22-question document to study risk factors for Q fever in Jordan; Muri et al. [7] have used a 30-question interview in Norway to study attitudes of sheep farmers; Morales-Pablos et al. [8] have used a 13-question document to evaluate risk factors for paratuberculosis in Mexico; Higino et al. [9] have used a 19-question document to assess risk factors for leptospirosis in Brazil; Delafosse et al. [10] have used a 35-question document to assess risk factors for cryptosporidiosis in France; Farkas and Hall [11] have used a 18-question document to study myiasis in Hungary. Use of questionnaires with small numbers of questions can lead in missing points and omitting collecting potentially valuable data during the field work, which would hinder correct analysis of the data at subsequent stages. Some of the questions asked in those questionnaires, have also been included in the present one (e.g., number and breed of animals in the farm; vaccination schedules, feeding regimes).

Use of similar questions in different research studies will also facilitate standardization of the work and easier comparison of the results between the studies. Questions from the present questionnaire can be combined with other ones, more specific and pertaining to the particular nature of a problem under study, which would be developed by researchers for specific projects. For example, in a field study regarding specifically reproductive management of sheep/goats, further relevant questions could be potentially added; examples include: in question 285, a description of the hormonal treatment (e.g., duration of intravaginal administration of progestogen sponges), and in question 290, the type of artificial insemination (e.g., intravaginal or intrauterine insemination) and the type of semen used (e.g., fresh or frozen semen). The above exemplify that specific additional questions can be formulated and added according to the particular needs of each occasion.

The present questionnaire can also be used as a model for recording farm details in cases of clinical work. Indeed, efficient history taking can be greatly facilitated by using a standardised questionnaire that could explore various issues and situations in the farm. The questionnaire can be handled by clinicians for collection of data in farms, in order to monitor changes in the situation with time. The usefulness of the questionnaire for such circumstances was corroborated by the unanimously positive comments of the clinicians and their request to have a copy for their own use in clinical work.

The recent establishment of EU-wide regulation regarding risk assessment in the food chain (which includes animal farms as the first step in the production of foods of animal origin) [12] underlines the usefulness of the present questionnaire, for collection and recording of detailed information in a structured way. Relevant national legislations also exist and the present questionnaire can also be adopted and applied in these approaches. For example, the ‘ClassyFarm’ is an Italian innovation and the result of a project commissioned by the Italian Ministry of Health, with the objective to improve and support the collaboration between farmers and the competent authorities of that country, in order to improve the safety and quality of products produced in animal farms. Under those circumstances, there is a potential to run the questionnaire to record and file detailed information in these farms. As part of assessments performed within the context of the above strategies, the questionnaire can be useful for recording in the field, storing the data and then evaluating the risk of the most frequent or probable problems in the participating farms.

Questionnaires with in-farm visits, such as the present investigation, have the advantage that they can provide an in-depth knowledge of the circumstances than telephone-, mail- or computer-based questionnaires [13]. This has become evident in the present work as farmers asked for clarifications during the interview, which would not have been possible if data had not been collected by personal visits. Further, a farm visit can be used also for collection of various samples, in order to obtain further data regarding the situation in a farm, depending on the specific purpose of a study. A significant disadvantage of in-farm surveys is the increased cost and time involved in collection of the data [13]; this can be frustrating in cases that the potential respondents refuse to accept the interview after arrival to the farm. The comments of the farmers indicated that a visit with a long questionnaire might not be always welcome, especially in periods of increased work-load at the farm (e.g., vaccination time, lambing season, harvesting period) [14].

The duration of the interview can be a factor that may adversely affect attitude of the farmers [15] and ultimately the quality of replies [16]. The duration of the interview can depend on the investigator and the respondent. The effect of the investigator was seen in the progressive reduction of the duration of the interviews. As the investigator presented the interview to more farmers, a small, but significant, reduction of the duration was evident. One may postulate that possibly, with time, the investigator could recite the questionnaire with less need to look into the questions, which ultimately contributed in the progressively reduced duration of the interviews.

Response latency, which is mirrored in the final duration of the interview, is the time necessary for a respondent to answer the questions and is considered to be associated with ‘the amount of information processing necessary to answer a question’ [17]. This was exemplified in the present study by farmers, who did not speak Greek as their primary language, taking significantly longer time to complete the interview. As these respondents did not ask for more clarifications than farmers who spoke Greek as their primary language, we postulate that the cause of the longer duration of the interview was possibly a latency in responding, rather than not fully understanding questions. Indeed, Wenz et al. [18] have reported that in face-to-face interviews respondents took more time to answer, but provided answers to more questions than in telephone-based interviews. Moreover, although it could be expected that older people would need more time to answer questions and that respondents with a higher educational level would be more familiar with answering questions, hence would need less time [19], no such effects were seen in this work. This can be the result of asking straightforward questions directly related to the daily professional circumstances and works of the respondents, coupled also with the fact that older farmers would be more experienced and proficient with professional issues, as well as more accustomed with management routine in their flocks/herds than younger, less experienced farmers.

The farmers were found to be positive and welcoming in the visits; the rate of refusals to an interview was negligible. Arrangement of the visit by the veterinarian, with whom the farmers were collaborating and whom they trusted, was a factor that obviously contributed. This also helped in practical ways, e.g., to arrange the visit at a time suitable for the farmer and to easily locate the farm at the countryside. A question that farmers often asked at the start of the visit was “*How long will it take?*”; this indicates the importance of the brevity of interviews for future survey work. The present results suggest that a 60- to 70-min-long interview would be near the longest period that farmers might accept.

Requests for clarifications by farmers were an important factor found to influence the duration of the questionnaire. Some of the points asked (e.g., embryo transfer, induction of lambing, colostrum bank) were the direct results of lack of relevant knowledge, as these are not applied frequently in small ruminant health management [20], hence farmers would not have heard of them at all.

During the initial assessment of the questionnaire, all farmers provided at least one inconsistent reply in the repeated interviews. Nevertheless, the high Cronbach’ coefficient alpha indicated an excellent internal consistency of the replies to the questionnaire [21]. In most cases, the inconsistencies referred to open questions, which needed a description or the provision of qualitative data from the respondent. The frequent lack of detailed records regarding production characteristics in farms may contribute further to providing erroneous replies during the interviews. The findings indicate that answers to open-type questions, which seek descriptive information, should be treated more cautiously than answers to dichotomous or multiple-choice questions. In order to cross-check and verify as much as possible of the information obtained by farmers during the interviews, details were also recorded for some of the questions by other investigators during the on-site visit, whilst some other details were collected from the accompanying veterinarians.

In the present study, the interviews were carried out consistently by the same investigator (DTL), who in fact performed the main investigation after an initial accustomisation and use of the questionnaire in farmers before the main study. This helped the investigator to familiarize themselves with the attitude of farmers and their way of responding to the questionnaire. Moreover, it minimized potential effects of the interviewer in the quality of data collected [22]. In cases of potential future use of the questionnaire in research work, it is, thus, recommended that initial small-scale field work is performed so that the future investigators familiarize themselves with the questionnaire and the approach of the farmers. As found in the present study, this might also help to decrease the duration of the interview. This, coupled with the performance of the interview by the same investigators, would contribute to increased data quality from the use of the questionnaire.

## 5. Conclusions

A detailed questionnaire to evaluate health management in small ruminant dairy farms has been developed, assessed and presented. The questionnaire includes 442 questions, arranged in seven sections. The questionnaire shows an excellent internal consistency as evidenced by a high Cronbach’s coefficient alpha. The mean duration of the interview was 63.6 min. Clarifications were often asked (by 61.5% of respondents) in 5.0% of the questions.

The questionnaire can be used for research work in the field, to record details in the farms under study. In accord with the needs of a particular study, it can be modified, by adding more specific questions or omitting others deemed to be less important. Moreover, it can also be used for routine monitoring purposes, as a useful means to record and maintain details of farms during clinical work. To the best of our knowledge, this is the most extensive and detailed relevant questionnaire available internationally for small ruminant farms.

## Figures and Tables

**Table 1:** Types of questions (n) in the questionnaire according to the various sections therein.

	Type of Question				
Section	Open	Multiple-Choice	Dichotomous	Scaling	Total
General	8	2	1	0	11
Infrastructure	39	17	63	0	119
Animals	43	4	38	0	85
Production characteristics	15	2	2	0	19
Health management	61	16	70	3	150
Nutrition	12	13	12	0	37
Human resources	12	1	8	0	21
Total	190	55	194	3	442

**Table 2:** Details of farms, in which interviews were performed.

Animal Species in the Farms	Management System ^1^				
	Intensive	Semi-Intensive	Semi-Extensive	Extensive	Total
Sheep	43	151	107	24	325
Goats	9	31	58	21	119
Total	52	182	165	45	444

^1^ Management system practiced in the farms was defined according to the European Food Safety Authority [3].

**Table 3:** Frequency of the various qualitative comments about the questionnaire, spontaneously made by 24 veterinarians, who attended the interviews.

Comment	No. of Veterinarians Who Made the Comment
Extensive and covering all relevant matters	14
Good	12
Useful for the small ruminant industry	9
Useful for practicing veterinarians	6
Very good	6

## Supplementary Materials

The following are available online at https://www.mdpi.com/2076-2615/10/9/1489/s1, Table S1: A detailed questionnaire for the evaluation of health management in sheep or goat dairy farms.

## Appendix A

Questions for which relevant details were recorded (additionally to the answers received during the interview) during a thorough on-site evaluation of all relevant areas in the farm during 444 visits.

**No.**	**Question**
014	Availability of a main building for animals
015	Availability of a dedicated building for lambs/kids
024	Availability of a milking parlour
025	Availability of a waiting area before the milking parlour
031	Availability of footbath
039	Availability of a ditch at the main entrance
040	Material of the walls
041	Material of the roof
043	Openings in the walls
044	Number of openings in the walls
045	Opening in the roof
047	Material of the floor
048	Availability of straw bedding
049	Availability of ventilators
051	Number of available ventilators
052	Availability of artificial lighting
054	Availability of milk replacer facilities
055	Availability of equipment for administration of milk replacer to lambs
056	Availability of milk heating facilities
057	Number of plastic teats available
058	Openings in the walls
059	Openings in the walls
060	Opening in the roof
068	Material of the floor
069	Type of milking system
070	Type of milking parlour
071	Number of animal positions in the parlour
072	Number of available milking units
074	Availability of facilities for milk yield measurement
075	Type of facilities for milk yield measurement
076	Availability of milk quality indicators
077	Availability of milk flow indicators
078	System pulsation rate
079	System pressure
080	Type of flow line
089	Availability of a milk tank
090	Availability of a mixer in the milk tank
091	Temperature in the milk tank
099	Availability of a feed mill
100	Availability of an automated feeding system
103	Availability of automatic water filling system in troughs
104	Availability of system for waste removal
113	Total number of feed troughs available
114	Type of feed troughs available
115	Total number of drinking troughs available
116	Type of drinking troughs available
322	Farm security
324	Availability of disinfectant at entrance ditch

## Appendix B

Sheep flocks or goat herds visited in the 13 administrative regions of Greece, for completion of the questionnaire.

**Administrative Region**	**No. of Sheep Flocks**	**No. of Goat Herds**
Attica	1	1
Central Greece	26	6
Central Macedonia	25	11
Crete	19	10
Eastern Macedonia and Thrace	34	16
Epirus	18	4
Ionian islands	3	1
North Aegean	19	3
Peloponnese	45	25
South Aegean	2	2
Thessaly	71	18
Western Greece	45	17
Western Macedonia	17	5
Total	325	119

## Appendix C

Questions for which relevant details were collected (additionally to the answers received during the interview) from the veterinarians, who had arranged the visits and had an association with the respective farms, during 444 visits.

**No.**	**Question**
260	Diseases of adult animals—mastitis: Total cases during the preceding season
261	Diseases of adult animals—mastitis: Sample collection and testing for diagnostic purposes
262	Diseases of adult animals—mastitis: Treatment
263	Diseases of adult animals—mastitis: Pharmaceuticals used for treatment
264	Diseases of adult animals—mastitis: Route for administration of antimicrobials
265	Diseases of adult animals—abortion: Total cases during the preceding season
266	Diseases of adult animals—abortion: Sample collection and testing for diagnostic purposes
267	Diseases of adult animals—abortion: Types of samples collected for testing
268	Diseases of adult animals—abortion: Pharmaceuticals used for treatment
269	Diseases of adult animals—abortion: Collection of aborted material for safe disposal
270	Diseases of adult animals—pregnancy toxaemia: Total cases during the preceding season
271	Diseases of adult animals—pregnancy toxaemia: Treatment performed
272	Diseases of adult animals—lameness: Total cases during the preceding season
273	Diseases of adult animals—lameness: Treatment performed
274	Diseases of adult animals—mange: Total cases During the preceding season
275	Diseases of adult animals—mange: Treatment performed
276	Diseases of adult animals—obstetrical cases: Total cases during the preceding season
277	Diseases of adult animals—obstetrical cases: Call for veterinary support
279	Diseases of young animals—respiratory problems: Total cases during the preceding season
280	Diseases of young animals—respiratory problems: Treatment performed
281	Diseases of young animals—respiratory problems: Pharmaceuticals used for treatment
282	Diseases of young animals—diarrhoea: Total cases during the preceding season
283	Diseases of young animals—diarrhoea: Treatment performed
284	Diseases of young animals—diarrhoea: Pharmaceuticals used for treatment
325	Vaccinations: Against *Chlamydia* infection
326	Description of schedule:
327	Vaccinations: Against *Toxoplasma* infection
328	Description of schedule:
329	Vaccinations: Against *Brucella* infection
330	Description of schedule:
331	Vaccinations: Against clostridial infection
332	Description of schedule:
333	Vaccinations: Against mastitis
334	Description of schedule:
335	Vaccinations: Against contagious agalactia
336	Description of schedule:
337	Vaccinations: Against bacterial respiratory infections
338	Description of schedule:
339	Vaccinations: Against orf
340	Description of schedule:
341	Vaccinations: Against paratuberculosis
342	Description of schedule:
343	Vaccinations: Against foot-rot
344	Description of schedule:
345	Administration of anthelmintics to sheep/goats in the farm
346	Administration of anthelmintics to all or only some animals in the farm at the same time
347	Timing of administration within the annual production cycle
348	Anthelmintics administered
349	Pharmaceutical form administered
352	Administration of ectoparasiticides to sheep/goats in the farm
353	Timing of administration within the annual production cycle
354	Ectoparasiticides administered
355	Pharmaceutical form administered
358	Administration of antiparasitics to dogs in the farm
359	Antiparasitics used
360	Pharmaceutical form administered

## Appendix D

Questions in which inconsistencies were recorded in the answers of 27 farmers, interviewed by two different persons two to three months apart.

**No.**	**Question**	**No. of Farmers Who Gave Inconsistent Answers**
065	(Milking parlour) Year of initial establishment	3
066	(Milking parlour) Year of most recent renovation	4
125	Private land grazed	6
128	Total surface of the cultivated land	9
141	Average age of culling ewes/does	13
143	Average annual replacement rate of ewes/does	14
180	Description of bioindications of rodents	5
186	Annual frequency of administration of rodenticides	7
202	Presence of nests within the farm buildings	4
217	Usual month of the start of the milking period	1
218	Usual month of the end of the milking period	3
219	Total milk quantity obtained during the preceding milking period	7
223	Total number of lambs/kids born during the preceding lambing season	9
224	Total number of lambs/kids sold during the preceding season	5
235	The two health problems in lambs/kids considered to be of the greater importance	7
239	The two health problems in adult animals considered to be of the greater importance	8
244	Routine overdosing (compared to dose prescribed) of pharmaceuticals	3
250	Total visits made annually by veterinarians to the farm during the preceding season	3
260	(Diseases of adult animals—mastitis) Total cases during the preceding season	5
265	(Diseases of adult animals—abortion) Total cases during the preceding season	6
276	(Diseases of adult animals—obstetrical cases) Total cases during the preceding season	2
279	(Diseases of young animals—respiratory problems) Total cases during the preceding season	6
282	(Diseases of young animals—diarrhoea) Total cases during the preceding season	5
316	Disposal of carcasses from dead animals	1
372	Annual frequency of foot care	2
393	Total quantity of hay consumed during the preceding season	7
408	Total quantity of finished feed (concentrate) consumed during the preceding season	10
422	Age	10
432	Personal opinion regarding occurrence of transmission of diseases from animals to the farmer or members of the family	1
433	Diseases, according to above, for which transmission occurred from animals	1

## Appendix E

Questions in which clarifications were asked by farmers during 444 interviews.

**No.**	**Question**	**No. of Farmers who Requested Further Explanations**
016	Availability of a dedicated lambing/kidding area	14
035	Proximity to industrial sites	7
076	Availability of milk quality indicators	38
077	Availability of milk flow indicators	7
080	Type of flow line	30
083	Type of system check-ups performed by technicians	19
098	Availability of a roller crusher	9
103	Availability of automatic water filling system in troughs	10
112	Availability of animal location identifiers	23
119	Availability of sensors for registration of environmental conditions	24
141	Average age of culling ewes/does	76
146	Criteria for selection of own animals as replacements	47
245	Use of laboratory diagnostic examinations	61
253	Evaluation of ammonia concentration within the buildings	36
287	Changes of rams/bucks into the ewes/does during the mating period	63
288	Castration of lambs/kids kept for fattening	5
289	Use of vasectomies	56
291	Use of embryo transfer	89
293	Nutritional modifications before the mating period	12
300	Induction of lambing	38
303	Lamb/kid fostering to female animals other than their dams	5
394	Plants included in hay consumed by animals	46
