# Supplementary material for: A Detailed Questionnaire for the Evaluation of Health Management in Dairy Sheep and Goats
Source: Animals (Basel). 2020 Aug 24;10(9):1489. doi: 10.3390/ani10091489 (PMC7552296; doi:10.3390/ani10091489)
Supplement: Supplementary file 1 [file animals-10-01489-s001.pdf]

animals

MDPI

Article

A Detailed Questionnaire for Evaluation of Health Management in Dairy Sheep and Goats

Daphne T. Lianou, Ioanna P. Chatziprodromidou, Natalia G.C. Vasileiou, Charalambia K. Michael, Vasia S. Mavrogianni, Antonis P. Politis, Nick G. Kordalis, Charalambos Billinis, Alexios Giannakopoulos, Elias Papadopoulos, Ilias Giannenas, Katerina S. Ioannidi, Angeliki I. Katsafadou, Dimitris A. Gougoulis, Delia Lacasta, Mariangela Caroprese, George C. Fthenakis

Supplementary material S1. A detailed questionnaire for the evaluation of health management in sheep or goat dairy farms.

No.	Question
**0. GENERAL**	
001	Farm reference (no.)
002	National identification of the farm (no.)
003	Name of the farmer (name)
004	Address (description)
005	Date of the visit (date)
006	Veterinarian(s) (name)
007	Geographical coordinates (geographical latitude & longitude)
008	Production type (dairy / meat / wool / reproduction / other)
009	Management system (EFSA classification: shepherding / intensive / semi-intensive / semi-extensive / extensive / very extensive / mixed)
010	Organic farm (yes / no)
011	Start date of the most recent lambing / kidding season (date)
**1. INFRASTRUCTURE**	
**1.1. General information**	
012	Year of the initial establishment of the farm (year)
013	Year of establishment at the present location (year)
014	Availability of a main building for animals (yes / no)
015	Availability of a dedicated building for lambs / kids (yes / no)
016	Availability of a dedicated lambing / kidding area (yes / no)
017	Number of pens in the lambing / kidding area (no.)
018	Accessory building(s) for animals (yes / no)
019	Availability of sheds (yes / no)
020	Number of available sheds (no.)
021	Availability of sheds for storage of concentrate feedstuffs (yes / no)
022	Availability of sheds for storage of roughage (yes / no)
023	Availability of sheds for storage of silage (yes / no)
024	Availability of a milking parlour (yes / no)
025	Availability of a waiting area before the milking parlour (yes / no)
026	Availability of personnel areas (yes / no)
027	Availability of area for veterinary works (yes / no)
028	Availability of an office (yes / no)
029	Availability of a lavatory (yes / no)
030	Drainage type (local drainage network / cesspit)
031	Availability of footbath (yes / no)
032	Availability of isolation facilities for animals (yes / no)
033	Availability of access road to the farm (yes / no)
034	Electricity source (electricity network / generator / solar cells / other)

035	Proximity to industrial sites (yes / no)
036	Proximity to industrial sites (distance)
037	Type of industrial sites near the farm (description)
038	Availability of waste processing facility (yes / no)
039	Availability of a ditch at the main entrance (yes / no)
**1.2. Buildings**	
**1.2.1. Main building**	
040	Material of the walls (bricks / plastered bricks / cement / wood / stone / plastic / straw / panel / tin / cinder blocks / other)
041	Material of the roof (bricks / plastered bricks / cement / wood / stone / plastic / straw / panel / tin / cinder blocks / tiles / other)
042	Dimensions of the building (length × width × height in m)
043	Openings in the walls (yes / no)
044	Number of openings in the walls (no.)
045	Opening in the roof (yes / no)
046	Orientation of the building (degrees)
047	Material of the floor (cement / soil / slatted wood / slatted metal / other)
048	Availability of straw bedding (yes / no)
049	Annual frequency of removal / clean-up of the straw bedding (no. of occasions)
059	Availability of ventilators (yes / no)
051	Number of available ventilators (no.)
052	Availability of artificial lighting (yes / no)
**1.2.2. Building for lambs / kids**	
053	Dimensions of the building (length × width × height in m)
054	Availability of milk replacer facilities (yes / no)
055	Availability of equipment for administration of milk replacer to lambs (yes / no)
056	Availability of milk heating facilities (yes / no)
057	Number of plastic teats available (no.)
058	Openings in the walls (yes / no)
059	Openings in the walls (no.)
060	Opening in the roof (yes / no)
061	Administration of milk replacer to lambs (yes / no)
062	Daily frequency of administration of milk replacer to lambs (no. of occasions)
063	Grouping of lambs for administration of the milk replacer (yes / no)
064	Criteria for grouping of lambs for administration of the milk replacer (description)
**1.2.3. Milking parlour**	
065	Year of initial establishment (year)
066	Year of most recent renovation (year)
067	Dimensions of the parlour (length × width × height in m)
068	Material of the floor (cement / tile / soil / other)
069	Type of milking system (mobile / non-mobile)
070	Type of milking parlour (fishbone / circular / linear parallel / linear one-sided / other)
071	Number of animal positions in the parlour (no.)
072	Number of available milking units (no.)
073	Provision of feed during milking (yes / no)
074	Availability of facilities for milk yield measurement (yes / no)
075	Type of facilities for milk yield measurement (individual / group)
076	Availability of milk quality indicators (yes / no)
077	Availability of milk flow indicators (yes / no)
078	System pulsation rate (no. s^{-1})
079	System pressure (no.)
080	Type of flow line (low / high)
081	Weekly frequency of check-ups of the system by farmer (no. of occasions)
082	Annual frequency of check-ups of the system by technicians (no. of occasions)
083	Type of system check-ups performed by technicians (dynamic / static)
084	Water cleaning of parlour after the milking sessions (yes / no)
085	Temperature of cleaning water (^{o}C)
086	Use of detergent for parlour cleaning after the milking sessions (yes / no)

087	Criteria for changing teatcups (description)
088	Annual frequency of changing teatcups (no. of occasions)
089	Availability of a milk tank (yes / no)
090	Availability of a mixer in the milk tank (yes / no)
091	Temperature in the milk tank (^{o}C)
092	Frequency of milk collection (description of the no. of times that the dairy company collects the milk)
093	Frequency of cleaning of milk tank (description of the occasions that the milk tank is routinely cleaned)
094	Date of the preceding collection of milk (description)
**1.3. Equipment**	
095	Availability of a crate (yes / no)
096	Availability of scales (yes / no)
097	Scale type available (large / portable)
098	Availability of a roller crusher (yes / no)
099	Availability of a feed mill (yes / no)
100	Availability of an automated feeding system (yes / no)
101	Availability of a silage distributor (yes / no)
102	Availability of a straw cutter (yes / no)
103	Availability of automatic water filling system in troughs (yes / no)
104	Availability of system for waste removal (yes / no)
105	Availability of bathing tank (yes / no)
106	Availability of a generator (yes / no)
107	Availability of a tractor (yes / no)
108	Availability of a truck (yes / no)
109	Availability of a pick-up (yes / no)
110	Animal identification system used (ear-tags / boli / necklace)
111	Availability of automatic animal identification system (yes / no)
112	Availability of animal location identifiers (yes / no)
113	Total number of feed troughs available (no.)
114	Type of feed troughs available (metal / wood / plastic)
115	Total number of drinking troughs available (no.)
116	Type of drinking troughs available (metal / wood / plastic)
117	Availability of refrigerators (yes / no)
118	Description of available refrigerators (domestic use-type refrigerators / professional use-type refrigerators / deep freezers)
119	Availability of sensors for registration of environmental conditions (yes / no)
120	Description of available sensors for registration of environmental conditions (description)
121	Practicing sharing of equipment with other farms (yes / no)
**1.4. Land**	
122	Grazing practiced (yes / no)
123	Total grazing land by the farm animals (acres)
124	Ownership of the grazing land (farmer / other private / public)
125	Private grazing land (acres)
126	Irrigation of the grazing land (yes / no)
127	Grazing of cultivated land (yes / no)
128	Total surface of the cultivated land (acres)
129	Plant types available in the grazing land (description)
130	Use of hydroponic facilities (yes / no)
**2. ANIMALS**	
**2.1. Small ruminants**	
131	Main animal species (sheep / goats)
132	Mixed farm (yes / no)
133	No. of ewes (no.)
134	No. of rams (no.)
135	No. of does (no.)
136	No. of bucks (no.)
137	Main breed of ewes / does (description)

138	Secondary breed of ewes / does (description)
139	Main breed of rams / bucks (description)
140	Secondary breed of rams / bucks (description)
141	Average age of culling ewes / does (years)
142	Average age of culling rams / bucks (years)
143	Average annual replacement rate of ewes / does (%)
144	Average annual replacement rate of rams / bucks (%)
145	Source of replacement animals (own animals / purchase)
146	Criteria for selection of own animals as replacements (description)
147	Criteria for selection of animals for purchase as replacements (description)
148	Purveyors of replacements to be purchased (description)
**2.2. Other domestic animals in the farm**	
149	Adult cattle (yes / no)
150	Adult cattle (no.)
151	Calves (yes / no)
152	Calves (no.)
153	Buffaloes (yes / no)
154	Buffaloes (no.)
155	Pigs (yes / no)
156	Pigs (no.)
157	Rabbits (yes / no)
158	Rabbits (no.)
159	Poultry (yes / no)
160	Poultry (no.)
161	Domestic birds (yes / no)
162	Domestic birds (no.)
163	Dogs (yes / no)
164	Dogs (no.)
165	Sheepdogs (yes / no)
166	Sheepdogs (no.)
167	Cats (yes / no)
168	Cats (no.)
169	Exotic animals (yes / no)
170	Exotic animals (no.)
171	Horses (yes / no)
172	Horses (no.)
173	Donkeys or mules (yes / no)
174	Donkeys or mules (no.)
175	Other animals (yes / no)
176	Other animals (no.)
**2.2.1. Rodents in the farm**	
177	Visual contacts of the farmer with rodents (yes / no)
178	Recognition of dead rodents in the farm grounds or within a radius of 500 m of the farm (yes / no)
179	Bioindications of rodents in the farm grounds or within a radius of 500 m of the farm (yes / no)
180	Description of bioindications of rodents (feed / faeces / other)
181	Recognition of rodents in feed troughs (yes / no)
182	Recognition of rodents in water troughs (yes / no)
183	Presence of rodents in fields within a radius of 2 km of the farm (yes / no)
184	Rodents identified within a radius of 2 km of the farm (description)
185	Administration of rodenticides (yes / no)
186	Annual frequency of administration of rodenticides (no. of occasions)
**2.3. Wildlife**	
**2.3.1. Wildlife mammals**	
187	Visual contacts of the farmer with wildlife mammals (yes / no)
188	Recognition of dead wildlife mammals in the farms grounds or within a radius of 500 m of the farm (yes / no)
189	Recognition of traces of wildlife mammals within a radius of 2 km of the farm (yes / no)
190	Recognition of wildlife mammals within a radius of 2 km of the farm (yes / no)

191	Recognition of damage to cultivations within a radius of 2 km of the farm (yes / no)
192	Population of wildlife mammals recognised within a radius of 2 km of the farm (no. of animals)
193	Wildlife mammals identified within a radius of 2 km of the farm (description)
194	Common grazing of sheep / goats with wildlife mammals (yes / no)
195	Species of wildlife mammals identified in common grazing (description)
**2.3.2. Avian wildlife**	
196	Visual contacts of the farmer with avian wildlife (yes / no)
197	Recognition of dead avian wildlife in the farms grounds or within a radius of 500 m of the farm (yes / no)
198	Bioindications of avian wildlife in the farms grounds or within a radius of 500 m of the farm (yes / no)
199	Description of bioindications of avian wildlife (bird calls / feathers / feed / eggs)
200	Recognition of avian wildlife in feed troughs (yes / no)
201	Recognition of avian wildlife in water troughs (yes / no)
202	Presence of nests within the farm buildings (yes / no)
203	Presence of eggs in such nests (yes / no)
204	Presence of chicks in such nests (yes / no)
205	Population of avian wildlife recognised within a radius of 2 km of the farm (no. of animals)
206	Avian wildlife identified (description)
**2.3.3. Hunting**	
207	Presence of hunters in the area around the farm within a radius of 2 km of the farm (yes / no)
208	Number of hunters in the area within a radius of 2 km of the farm (no.)
209	Weekly frequency of the presence of hunters in the area (no. of daily occasions)
210	Description of hunted avian species (description)
211	Description of hunted mammalian species (description)
212	Distance from the farm that hunting activity occurs (km)
213	Months during which hunting activity occurs (description)
214	Hunting activity of the farmer (yes / no)
215	Length of hunting activity of the farmer (no. of years)
**3. PRODUCTION CHARACTERISTICS**	
216	Month of the start of the lambing / kidding season (description)
217	Usual month of the start of the milking period (description)
218	Usual month of the end of the milking period (month)
219	Total milk quantity obtained during the preceding milking period (litres)
220	Average fat content in milk during the preceding milking period (%)
221	Average protein content in milk during the preceding milking period (%)
222	Average somatic cell counts in milk during the preceding milking period (no.)
223	Total number of lambs / kids born during the preceding lambing season (no.)
224	Total number of lambs / kids sold during the preceding season (no.)
225	Average age of lambs / kids at slaughter (days)
226	Average live bodyweight of lambs / kids at slaughter (kg)
227	Average carcass weight of these at slaughter (kg)
228	Total number of ewe- or ram-lambs / doelings or bucklings sold during the preceding period (no.)
229	Average carcass weight of these at slaughter (kg)
230	Record keeping (yes / no)
231	Type of records kept (paper / electronic)
232	Local manufacturing of dairy products (yes / no)
233	Objective of local manufacturing of dairy products (sale / home consumption)
234	Types of dairy products in local production (description)
**4. HEALTH MANAGEMENT**	
**4.1. Health parametres**	
235	The two health problems in lambs / kids considered to be of the higher importance (description)
236	Total cases of these two health problems in lambs / kids during the preceding season (no.)
237	The two health problems in replacement animals considered to be of the higher importance (description)
238	Total cases of these two health problems in replacement animals during the preceding season (no.)
239	The two health problems in adult animals considered to be of the higher importance (description)
240	Total cases of these two health problems in adult animals during the preceding season (no.)

241	Total deaths, of any cause, in adult animals during the preceding season (no.)
242	Collaboration with a veterinarian (yes / no)
243	Means of calculating live bodyweight for the administration of pharmaceutical products (weighing / estimation)
244	Routine overdosing (compared to dose prescribed) of pharmaceuticals (yes / no)
245	Use of laboratory diagnostic examinations (yes / no)
246	In samples of milk (yes / no)
247	In samples of blood (yes / no)
248	In samples of faeces (yes / no)
249	Laboratory diagnostic examinations performed in these samples (description)
250	Total visits made annually by veterinarians to the farm during the preceding season (no.)
251	Reasons for the visits of the veterinarians (description)
252	Maintenance of prescribed withdrawal periods after administration of pharmaceuticals (yes / no)
253	Evaluation of ammonia concentration within the buildings (yes / no)
254	Animal deaths from attacks by wildlife animals (yes / no)
255	Total number of animal deaths by wildlife animals during the preceding season (no.)
256	Species of wildlife animals that caused animal deaths during the preceding season (description)
257	Animal deaths from natural disasters (yes / no)
258	Total number of animal deaths from natural disasters during the preceding season (yes / no)
259	Description of natural disasters that caused animal deaths (description)
**4.2. Health problems**	
**4.2.1.a. Diseases of adult animals – mastitis**	
260	Total cases during the preceding season (no.)
261	Sample collection and testing for diagnostic purposes (yes / no)
262	Treatment (yes / no)
263	Pharmaceuticals used for treatment (description)
264	Route for administration of antimicrobials (systematically / intramammary)
**4.2.1.b. Diseases of adult animals – abortion**	
265	Total cases during the preceding season (no.)
266	Sample collection and testing for diagnostic purposes (yes / no)
267	Types of samples collected for testing (description)
268	Pharmaceuticals used for treatment (description)
269	Collection of aborted material for safe disposal (yes / no)
**4.2.1.c. Diseases of adult animals – pregnancy toxaemia**	
270	Total cases during the preceding season (no.)
271	Treatment performed (description)
**4.2.1.d. Diseases of adult animals – lameness**	
272	Total cases during the preceding season (no.)
273	Treatment performed (description)
**4.2.1.e. Diseases of adult animals – mange**	
274	Total cases during the preceding season (no.)
275	Treatment performed (description)
**4.2.1.f. Diseases of adult animals – obstetrical cases**	
276	Total cases during the preceding season (no.)
277	Call for veterinary support (yes / no)
278	Person who performed manipulations (veterinarian / farmer / non-veterinary staff member)
**4.2.2.a. Diseases of young animals – respiratory problems**	
279	Total cases during the preceding season (no.)
280	Treatment performed (description)
281	Pharmaceuticals used for treatment (description)
**4.2.2.b. Diseases of young animals – diarrhoea**	
282	Total cases during the preceding season (no.)
283	Treatment performed (description)
284	Pharmaceuticals used for treatment (description)
**4.3. Management practices**	
285	Reproductive management (no hormonal control / administration of melatonin / administration of progestagens)
286	Duration of mating period (unlimited period / defined time-schedule)

287	Changes of rams / bucks into the ewes / does during the mating period (yes / no)
288	Castration of lambs / kids kept for fattening (yes / no)
289	Use of vasectomies (yes / no)
290	Use of artificial insemination (yes / no)
291	Use of embryo transfer (yes / no)
292	Use of ultrasound for pregnancy diagnosis (yes / no)
293	Nutritional modifications before the mating period (yes / no)
294	Nutritional modifications before the lambing period (yes / no)
295	Beginning of the mating period for ewes / does (month)
296	End of the mating period for ewes / does (month)
297	Beginning of the mating period for ewe-lambs and doelings (month)
298	End of the mating period for ewe-lambs and doelings (month)
299	Grouping of pregnant females during the final stage of pregnancy (yes / no)
300	Induction of lambing (yes / no)
301	Newborn care and specific monitoring (yes / no)
302	Maintenance of a colostrum bank (yes / no)
303	Lamb / kid fostering to female animals other than their dams (yes / no)
304	Reasons for doing this practice (description)
305	Administration of a lamb- / kid-specific diet (yes / no)
306	Age for lamb / kid removal from their dams (days)
307	Age of weaning of lambs / kids (days)
308	Daily number of milking sessions (no.)
309	Method for drying-off at the end of the lactation period (abrupt / progressive)
310	Duration of the dry-period (months)
311	Seasonal transfer of animals to other site (yes / no)
312	Means of animal transfer between sites (description)
313	Distance between sites (km)
314	Nights out during transfer between sites (yes / no)
315	Post-mortem examination of animals that die (yes / no)
316	Disposal of carcasses from dead animals (incineration / burying / feeding to dogs / feeding to birds / drop-off away)
317	Reporting to the farming insurance agency (yes / no)
318	Compensation by the farming insurance agency (yes / no)
319	Manure management (spread to fields / sale / disposal / feed to animals / biogas production)
320	Security presence at the farm (yes / no)
321	Duration of security attendance (continuous / morning & night visits / once daily visit / other)
322	Farm security (light wire fence / strong wire fence / stoned wall / wooden wall / alarm / other / no security)
323	Recording of vehicles entering into the farm (yes / no)
324	Availability of disinfectant at entrance ditch (yes / no)
**4.4. Vaccinations**	
325	Against *Chlamydia* infection (yes / no)
326	Description of schedule:
327	Against *Toxoplasma* infection (yes / no)
328	Description of schedule:
329	Against *Brucella* infection (yes / no)
330	Description of schedule:
331	Against clostridial infection (yes / no)
332	Description of schedule:
333	Against mastitis (yes / no)
334	Description of schedule:
335	Against contagious agalactia (yes / no)
336	Description of schedule:
337	Against bacterial respiratory infections (yes / no)
338	Description of schedule:
339	Against orf (yes / no)
340	Description of schedule:
341	Against paratuberculosis (yes / no)

342 Description of schedule:
343 Against foot-rot (yes / no)
344 Description of schedule:

4.5. Administrations of antiparasitics

4.5.1.a. Antiparasitic administrations – Anthelmintic treatments to small ruminants

345 Administration of anthelmintics to sheep / goats in the farm (yes / no)
346 Administration of anthelmintics to all or only some animals in the farm at the same time (all / some)
347 Timing of administration within the annual production cycle (before the mating season / at the beginning of dry-period / at the final stage of pregnancy / 1st-2nd month of the lactation period / 3rd-6th month of the lactation period)
348 Anthelmintics administered (description)
349 Pharmaceutical form administered (description)
350 Use of environmental applications for helminth control (yes / no)
351 Which ones (description)

4.5.2. Antiparasitic administrations – Ectoparasiticide treatments to small ruminants

352 Administration of ectoparasiticides to sheep / goats in the farm (yes / no)
353 Timing of administration within the annual production cycle (before the mating season / at the beginning of dry-period / at the final stage of pregnancy / 1st-2nd month of the lactation period / 3rd-6th month of the lactation period)
354 Ectoparasiticides administered (description)
355 Pharmaceutical form administered (description)
356 Use of environmental applications for ectoparasite control (yes / no)
357 Which ones (description)

4.5.3. Antiparasitic administrations – Antiparasitic treatments to dogs in the farm

358 Administration of antiparasitics to dogs in the farm (yes / no)
359 Antiparasitics used (description)
360 Pharmaceutical form administered (description)

4.6. Other health management practices

361 Application of disinfections in the farm (yes / no)
362 Annual frequency of systemic disinfections in the farm (no. of occasions)
363 Administration of oxytetracycline to the pregnant animals (yes / no)
364 Administration of selenium to pregnant animals (yes / no)
365 Administration of selenium to newborn animals (yes / no)
366 Administration of 'dry-ewe' treatment at the end of the lactation period (yes / no)
367 Use of teat disinfection before milking (yes / no)
368 Use of teat disinfection after milking (yes / no)
369 Use of teat spraying after milking (yes / no)
370 Weekly frequency of changing teat disinfectant in the cup (no. of occasions)
371 Foot care (yes / no)
372 Annual frequency of foot care (no. of occasions)
373 Shearing (yes / no)
374 Shearing equipment used (shearing shears / shearing machine)
375 Recording of births – maintenance of a lambing book (yes / no)
376 Disinfection of navel stumps in newborns (yes / no)
377 Tail docking in newborns (yes / no)
378 Routine administration of antimicrobials in newborns (yes / no)
379 Antimicrobials administered (description)
380 Maintenance of quarantine period for new animals into the farm (yes / no)
381 Isolation of sick animals (yes / no)

4.7. Vectors

382 Presence of spots suitable for reproduction of vectors (yes / no)
383 Types of spots identified (muddy spots inside or outside the buildings, near the water troughs / spots of wet manure / ditches with manure)
384 Distance of spots from farm (>50 m / 50-500 m / >500 m)

5. NUTRITION

385 Grazing (yes / no)
386 Duration of grazing during the winter (no. of months)

387	Distance from farm of area grazed during the winter (km)
388	Duration of grazing during the summer (no. of months)
389	Distance from farm of area grazed during the summer (km)
390	Type of graze area (meadow / wetland / scrub pasture / hay / forest / other)
391	Common grazing for sheep and goats (yes / no)
392	Provision of hay as fodder to animals (yes / no)
393	Total quantity of hay consumed during the preceding season (tonnes)
394	Plants included in hay consumed by animals (description)
395	Hay type (dried plant / pelleted)
396	Origin of hay (own production / purchase)
397	Provision of straw to animals (yes / no)
398	Provision of silage to adult animals (yes / no)
399	Provision of silage to young animals (yes / no)
400	Total quantity of silage consumed during the preceding season (tonnes)
401	Origin of silage (own production / purchase)
402	Provision of finished feed to animals (yes / no)
403	Origin of silage (own production / purchase)
404	Provision of finished feed (concentrate) to animals throughout the year (yes / no)
405	Finished feed (concentrate) form provided to adult animals (mashed / pellets / flakes / other)
406	Provision of finished feed (concentrate) to young animals (yes / no)
407	Finished feed (concentrate) type provided to young animals (mashed / pellets / flakes / other)
408	Total quantity of finished feed (concentrate) consumed during the preceding season (tonnes)
409	Total quantity of raw materials purchased during the preceding season (tonnes)
410	Raw materials used by the farm in the diets (corn / wheat / barley / bran / soyabean meal / cottonmeal / sunflower meal / fats / salt / phosphate salts / magensium salts / limestone / trace minerals / water soluble vitamins / fat soluble vitamins)
411	Raw materials purchased by the farm for use in the diets (corn / wheat / barley / bran / soyabean meal / cottonmeal / sunflower meal / fats / salt / phosphate salts / magensium salts / limestone / trace minerals / water soluble vitamins / fat soluble vitamins)
412	Premix purchase for use in the diets (yes / no)
413	Nutrient content in finished feed (description)
414	Feed change in animals (abrupt / progressive)
415	Water source (local water board / drilling / other source)
416	Water provision to animals (water troughs / tank / water collectors / fountains / rivers / lakes / other)
417	Use of laboratory examinations for quality testing of feeds and raw material (yes / no)
418	Laboratory examinations used (description)
419	Use of laboratory examinations for quality testing of water (yes / no)
420	Laboratory examinations used (description)
421	Person responsible for nutritional management (farmer / nutritionist / animal scientist / veterinarian / other)
**6. HUMAN RESOURCES**	
**6.1. Farmer**	
422	Age (years)
423	Previous animal farming experience (yes / no)
424	Length of previous animal farming experience (years)
425	General education (yes / no)
426	General education (description)
427	Professional education (yes / no)
428	Professional education (description)
429	Primary language spoken (description)
430	Farmer by profession (yes / no)
431	Daily period of presence in the farm (hours)
432	Marital status (description)
**6.2. Public health**	
433	Personal opinion regarding occurrence of transmission of diseases from animals to the farmer or members of the family (yes / no)
434	Diseases, according to above, for which transmission occurred from animals (description)

**6.3. Family**	
435	Work of family members in the farm (yes / no)
436	Family tradition in farming (yes / no)
437	Total members of the family (no.)
**6.4. Staff**	
438	Ethnicity (Greek local / Greek from other part of the country / non-Greek)
439	Nationality of non-Greek nationals (description)
440	Age (years)
441	Previous farming experience (yes / no)
442	Length of previous animal farming experience (years)

© 2020 by the authors. Published under the terms and conditions of the Creative Commons Attribution (CC BY) license (http://creativecommons.org/licenses/by/4.0/).
